# Participation and autonomy five years after stroke: A longitudinal observational study

**DOI:** 10.1371/journal.pone.0219513

**Published:** 2019-07-08

**Authors:** Annie Palstam, Astrid Sjödin, Katharina Stibrant Sunnerhagen

**Affiliations:** Department of Clinical Neuroscience, Institute of Neuroscience and Physiology, Sahlgrenska Academy, University of Gothenburg, Gothenburg, Sweden; University of Ioannina School of Medicine, GREECE

## Abstract

**Objective:**

Stroke is the second most common cause of disability in the world. The purpose of this study was to evaluate the participation and autonomy of persons with stroke, five years after a stroke, and to explore potential associations between factors and perceived restrictions in participation and autonomy.

**Methods:**

This five-year follow-up survey study included individuals diagnosed with a first-time stroke during 2009–2010, in Gothenburg. The survey included the Impact of Participation and Autonomy-questionnaire (IPA-E), which comprised five domains: Autonomy Indoor, Family Role, Autonomy Outdoor, Work & Education, and Social Life & Relationships. Logistic regression analyses were used to analyze factors associated with participation restrictions.

**Results:**

At 5 years after a stroke, 457 patients were alive; of these, 281 responded to the follow-up survey. Participation restrictions were most pronounced in the IPA-E domains of Autonomy Outdoors, Work/Education, and Social Life and Relationships. In contrast, restrictions were less pronounced in the IPA-E domains of Autonomy Indoors and Family Role. Severe stroke, older age, and female sex predicted participation restrictions at five years after a stroke. Participation restrictions were partly explained by feelings of depression at five years after stroke. Problems associated with participation restrictions were most frequently observed in the areas of mobility, leisure, and help/support from other people.

**Conclusion:**

This study showed that participation and autonomy were restricted among persons with stroke at five years after the stroke. The domains perceived as most restricted were those that required high levels of physical, social, and cognitive abilities.

## Introduction

Stroke is a global health issue of major proportions; it is the second most common cause of death and disability in the world [1, 2]. Indeed, increasing numbers of people are affected by stroke, and the number that remains disabled from stroke is growing [1]. Stroke is a chronic condition that can cause long-term disability [3]. An important part of rehabilitating people with disabilities is participation. Involvement in different areas of life, such as work, family, and community is vital to an individual’s well-being and self-identity [4, 5]. Participation is influenced by different aspects of health. Complex interactions between those aspects can explain the consequences of the individual’s health condition, whether it is a disease or a disorder. Another important aspect of rehabilitation is autonomy. Autonomy includes the right to take part in the decision-making that concerns one’s health. Autonomy is considered necessary to achieve full participation in life [6].

To date, several studies have reported restricted levels of participation at 6 months to 4 years after a stroke [7–9]. Limitations were experienced mostly in the areas of physical independence, mobility, and occupation/hobbies. The factors that contributed to participation restrictions were older age, physical impairment due to stroke [9–11], anxiety, depression [8–13], female sex [10, 13], and severe stroke [13].

Participation and autonomy in life after a stroke have been related to the degree of physical ability, emotional health, cognitive health, and access to health care services [9, 14–17]. Patients experienced a high degree of participation in the activities of daily living, getting around the house, and activities regarding social relations [15, 17, 18]. In contrast, patients experienced restrictions in participating in activities outdoors and, in some cases, in activities regarding family life [15, 17]. Furthermore, some studies have reported that restrictions in participation were considered a major problem in areas regarding financial dealings, family roles, and the ability to help other people [15, 19].

Thus, different levels of participation after a stroke have been reported in a number of studies, and several explanatory factors have been suggested. However, to our knowledge, few studies have investigated how participation and autonomy were affected in the long term after a stroke.

### Objective

The present study aimed to investigate how participation and autonomy were affected at five years after a stroke and to explore factors that might be associated with perceived restrictions in participation and autonomy.

## Methods

### Study design

This longitudinal observational study included patients recruited for the Stroke Arm Longitudinal study at the University of Gothenburg–extended study (the SALGOT-extended study) [20–22]. The study was approved by the Regional Ethics Committee in Gothenburg, Sweden, on June 5^th^ 2013 (Reference number 225–08, T801-10, and 400–13).

### Participants and setting

This study included patients with first ever strokes, diagnosed according to the International Classification of Diseases, according to the following codes, I61: intracerebral hemorrhage, I63: ischemic stroke, or I60: subarachnoidal hemorrhage. All patients were treated at Sahlgrenska University Hospital, Sahlgrenska, Gothenburg, Sweden. Eligible patients were ≥18 years of age and resided in the Gothenburg urban area. Patients were included consecutively between February 4^th^, 2009 and December 2^nd^, 2010 into the SALGOT-extended at the University of Gothenburg [20, 22]. A total of 725 patients were included in the cohort. Five years later, a survey was mailed to the survivors (n = 457) to explore the long-term consequences of stroke with a set of questionnaires. After two reminders, questionnaires that had not been returned were considered missing. A total of 281 individuals completed and returned the postal survey, and thus, these patients were included in the present study.

### Data collection

Baseline patient characteristics, including age, sex, and stroke type, were collected from medical charts. Stroke severity was assessed at admittance to the hospital, based on the National Institute of Health Stroke Scale (NIHSS, 0–42, lower scores indicated lower severity). For patients with subarachnoid hemorrhages, stroke severity was assessed with the Hunt and Hess scale (H&H, 1–5, lower scores indicated lower severity) [23].

The five-year follow-up survey included questions on living circumstances, the Impact on Participation and Autonomy-English version (IPA-E) questionnaire, and the Stroke Impact Scale. The IPA-E was a generic questionnaire developed to assess the perceived impact on participation and autonomy in everyday life for people with chronic diseases [14, 24, 25]. A Swedish version was used in this study. The IPA-E included 41 items, each rated 0–4, where a lower score indicated a better state. The 41 items were categorized into five domains, including autonomy indoors, family role, autonomy outdoors, social life/relationships, and work/education. In addition, the IPA-E included nine questions on whether the impact on participation was perceived as problematic (rated 0–2, where a lower score indicated less problems).

The Stroke Impact Scale (SIS; range: 0–100, a higher score indicated a better state), was used to assess self-perceived recovery [26]. For statistical analyses, the scale was divided into 4 categories: scores 0–24, 25–49, 50–74, and 75 to 100.

### Statistics

The statistical analyses were performed with the IBM Statistical Package for Social Sciences (SPSS) version 22, for Windows. All tests were two-sided, and p-values <0.05 were considered significant.

Dropout analyses were conducted to control for differences in baseline characteristics between participants that responded and those that did not respond to the questionnaires. Dropout analyses were also conducted to control for IPA-domains with more than 25% missing data. The Chi-2 test was performed to compare categorical variables between groups. The Mann-Whitney-U test was performed to compare continuous variables between groups. When any domain of the IPA-E showed a response rate less than 75%, it was eliminated from further analysis.

For further statistical analyses, NIHSS and H&H data were merged into dichotomous variables: very mild stroke (NIHSS = 0–2 and H&H = I) and mild- severe stroke (NIHSS = 3–46 and H&H = II-V).

Logistic regression analyses were performed to estimate the potential value of baseline variables for predicting restricted participation and autonomy at five years after a stroke. We also used logistic regression to analyze problems related to the perceived restrictions in participation and autonomy, for each IPA domain separately. Furthermore, logistic regression was used to analyze factors potentially associated with perceptions of restricted participation and autonomy at five years after stroke. To perform the logistic regression analyses, the IPA-E domain scores were dichotomized into “restricted participation and autonomy” (scores = 1–4) and “unrestricted participation and autonomy” (score = 0). The score that described the problems experienced was dichotomized into “no problems experienced” (score = 0) and “problems experienced” (scores = 1–2). Univariate logistic regression was conducted to analyze potential predictors for each of the IPA-domains and for the ‘Problems experienced’ categories. Variables with p-values ≤0.25 in the univariate analysis were accepted for inclusion into the multivariable logistic regression analyses. Independent variables were tested for collinearity. The multivariable logistic regression analyses were executed in backward conditional mode. P-values <0.05 were considered significant. Lastly, the variables rejected from the univariate logistic regression analyses were reinserted, one by one, into the final model to disclose any significance. Again, p-values <0.05 were considered significant. In each model, independent variables were evaluated with odds ratios (OR) and 95% confidence intervals (CI). A receiver operating characteristics (ROC) curve was calculated for each IPA-domain. An area under the ROC curve ≥0.7 was considered acceptable.

## Results

### Clinical characteristics:

A total of 281 individuals were included in this five-year follow-up survey. The mean age at the time of stroke was 65.4 years (SD 13.5). The majority (61%) of the population was male. Ischemic stroke was the most common type of stroke (78%). Thrombolysis was performed in 26 individuals, and 9 individuals received thrombectomy. More than half of the respondents had experienced a very mild stroke (63%) and only 7% had experienced a severe stroke. The group with subarachnoid hemorrhages had the same characteristics: the majority had low scores on the H&H (Table 1). Data on stroke severity was missing for 29 individuals.

**Table 1:** Characteristics of the study population (n = 281).

Baseline characteristics	Mean (SD)	n (%)
Age at time of stroke, years	65.4 (13.5)	
Sex, female		110 (39.1)
Stroke type:		
*Ischemic stroke*		218 (77.6)
*Intracerebral hemorrhage*		36 (12.8)
*Subarachnoid hemorrhage*		27 (9.6)
Stroke severity (NIHH and H&H combined):		
*Very mild*		170 (67.7)
*Mild to severe*		81 (32.3)
Characteristics at five-year follow up	Mean (SD)	n (%)
Time to five-year follow up, years	4.9 (0.5)	
Age at the time of follow-up survey, years	69.8 (13.4)	
Experienced stroke more than once		43 (15.4)
Current living conditions:		
*Own residence*		269 (95.7)
*Assisted living*		5 (1.8)
*Nursing home*		6 (2.1)
*Other*		1 (0.4)
Living alone, yes		102 (37)
Have you returned to work?		
*Yes*		61 (66.3)
*Yes*, *part time*		13 (14.1)
*No*, *I didn’t work prior to stroke*		4 (4.3)
*No*, *but I am planning to*		3 (3.3)
*No*		11 (12.0)
Do you feel supported?		
*I don’t need support*		170 (60.7)
*Yes*, *I do*		85 (30.4)
*I don’t want any support*		9 (3.2)
*No*, *but I need support*		10 (3.6)
Do you feel depressed?		
*No*, *almost never*		129 (45.9)
*Sometimes*		103 (36.7)
*Often*		30 (10.7)
*All the time*		8 (2.8)
How mobile are you?		
*I move independently indoors and outdoors*		248 (88.3)
*I move independently indoors*, *but not outdoors*		21 (7.5)
*I can´t move without assistance*		8 (2.8)
How do you estimate your recovery? (0–100)		
*< 25*		12 (4.6)
*25–49*		22 (8.4)
*50–74*		67 (25.6)
*≥75*		161 (61.5)

*Abbreviations*: *NIHSS = National Institute of health Stroke Scale*, *H&H = Hunt and Hess*, *NIHH very mild = NIHSS 0–2 + H&H grade I*, *NIHH mild to severe = NIHSS 3–46 + H&H II-V Missing data*: *Stroke severity n = 29*, *Experienced a stroke more than once n = 1*, *Living alone n = 5*, *Have you returned to work n = 189*, *Do you feel supported n = 1*, *Do you feel depressed n = 9*, *How mobile are you n = 4*, *How do you estimate your recovery n = 19*.

The dropout analysis showed no significant differences in age or stroke severity between those that dropped out and those that completed the questionnaire. However, significantly more women than men failed to return the follow-up questionnaire (p<0.001).

### Five-year follow-up

Nearly everyone in the study population lived at home. The majority shared households with at least one individual (spouse/children/sibling or other, Table 1). Out of the participants that were of working age at follow-up (n = 93), about 80% returned to work, either full-time or part-time, within the five-year period after the stroke. A large proportion of the study participants reported feeling socially supported. However, feelings of depression were reported in more than half of the study population. Most study participants (88.3%) reported that they could move independently indoors and outdoors, without assistance. About 61% of participants considered themselves to have recovered to the 75% level, or higher (scale 0 to 100; Table 1).

### Participation and autonomy at five years after a stroke

Restricted participation (i.e., poor or very poor participation/autonomy) was perceived most frequently in the domains of Autonomy Outdoors and Work and Education (Table 2). Conversely, restricted participation was perceived least in the domains of Autonomy Indoors, Family Role, and Social Life and Relationships (Table 2). The highest standardized mean scores, which indicated more severely restricted participation and autonomy, were found in the domains of Autonomy Outdoors, Work and Education, and Social Life and Relationships.

**Table 2:** Participation and autonomy at five years after a stroke for each IPA domain.

IPA Domains:	Mean^i^ (SD); Median (range)	Very good n (%)	Good/Fair n (%)	Poor/Very Poor n (%)
**Autonomy Indoors**	0.5 (0.8); 0 (0–4)	186 (72)	59 (23)	14 (5)
**Family Role**	0.8 (0.9); 0 (0–3)	176 (69)	47 (18)	32 (13)
**Autonomy Outdoors**	1 (1); 1 (0–4)	137 (53)	73 (28)	50 (19)
**Social Life/Relationships**	0.9 (0.8); 1 (0–3)	143 (55)	91 (35)	27 (10)
**Work/Education**	1 (1); 0 (0–4)	58 (59)	23 (23)	18 (18)

^*i*^ *= Standardized mean*

*Missing data*: *Autonomy indoors n = 22*, *Family role n = 26*, *Autonomy outdoors n = 21*, *Social life/Relationships n = 20*, *Work/Education n = 182*

**Fig 1:**
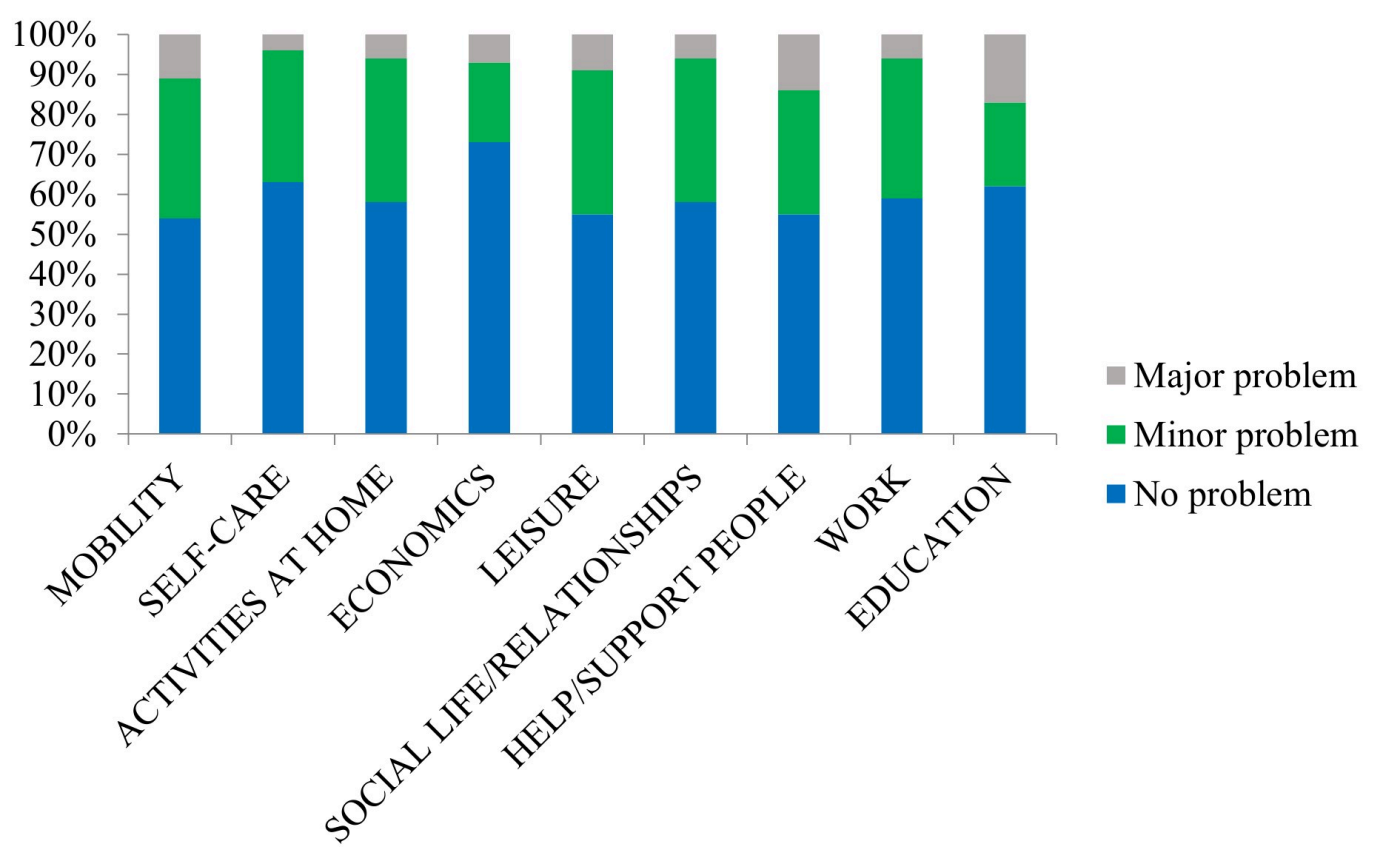
Self-perceived problems due to restrictions in participating in different life areas. Missing data: mobility n = 21, self-care n = 23, activities at home n = 28, economics n = 27, leisure n = 21, Social life n = 24, work n = 182, education = 234, help n = 30.

The problems experienced due to participation restrictions in different areas of life are shown in Fig 1. The highest frequencies of problems experienced were found in the life areas of mobility, leisure, and helping/supporting other people.

### Predictors of participation restrictions at five years after a stroke

The multivariate regression models for each IPA domain are presented in detail in Table 3. We found that older age, severe stroke, and female sex were independent predictors of perceived participation restrictions at five years after a stroke, in at least one IPA domain.

**Table 3:** Predictors of participation restrictions at five years after a stroke.

Predictor models	B (S.E.)	p-value	OR (95% CI)	AUC (95% CI)
**Autonomy Indoors:**				
Age	0.074 (0.015)	<0.001	1.077 (1.045–1.110)	0.747 (0.677–0.817)
Stroke Severity	0.710 (0.334)	0.034	2.033 (1.056–3.915)	
**Family Role:**				
Sex	0.776 (0.320)	0.015	2.173 (1.160–4.069)	0.753 (0.686–0.820)
Age	0.059 (0.015)	<0.001	1.061 (1.030–1.092)	
Severity	0.992 (0.332)	<0.001	2.696 (1.405–5.172)	
**Autonomy Outdoors:**				
Age	0.052 (0.012)	<0.001	1.054 (1.029–1.079)	0.719 (0.653–0.786)
Stroke Severity	1.046 (0.306)	0.001	2.847 (1.563–5.189)	
**Social Life/Relationship:**				
Age	0.024 (0.011)	0.024	1.025 (1.003–1.046)	0.631 (0.559–0.702)
Stroke Severity	0.725 (0.288)	0.012	2.065 (1.173–3.634)	
**Work/Education:**				
Stroke Severity	1.276 (0.501)	0.011	3.583 (1.342–9.567)	0.628 (0.505–0.751)

*Missing data*: *Autonomy indoors n = 52*, *Family role n = 55*, *Autonomy outdoors n = 51*, *Social life/Relationships n = 48*, *Work/Education n = 194*

### Explanatory factors for participation restrictions at five years after a stroke

The multivariate regression models for each IPA domain are presented in detail in Table 4. We found that, at five years after a stroke, older age, severe stroke, female sex, and feeling depressed contributed to participation restrictions in at least one IPA domain.

**Table 4:** Explanatory factors for participation restrictions at five years after a stroke.

Explanatory models	B (S.E.)	p-value	OR (95% CI)	AUC (95% CI)
**Autonomy Indoors:**				
Current age	0.079 (0.018)	<0.001	1.082 (1.047–1.117)	0.821 (0.760–0.881)
Feelings of depression	2.259 (0.428)	<0.001	9.973 (4.346–22.882)	
**Family Role:**				
Stroke severity	1.097 (0.361)	0.002	2.996 (1.476–6.083)	0.807 (0.743–0.872)
Current age	0.068 (0.016)	<0.001	1.070 (1.037–1.104)	
Feelings of depression	1.561 (0.369)	<0.001	4.761 (2.309–9.819)	
**Autonomy Outdoors:**				
Stroke severity	0.924 (0.344)	0.007	2.518 (1.284–4.938)	0.817 (0.760–0.873)
Current age	0.059 (0.014)	<0.001	1.067 (1.033–1.090)	
Feelings of depression	1.953 (0.331)	<0.001	7.051 (3.685–13.492)	
**Social Life/Relationship:**				
Stroke severity	0.668 (0.318)	0.036	1.950 (1.045–3.639)	0.743 (0.678–0.808)
Current age	0.023 (0.011)	0.040	1.024 (1.001–1.047)	
Feelings of depression	1.630 (0.305)	<0.001	5.103 (2.804–9.286)	
**Work/Education:**				
Feelings of depression	2.107 (0.504)	<0.001	8.222 (3.065–22.059)	0.772 (0.674–0.870)

*Missing data*: *Autonomy indoors n = 62*, *Family role n = 65*, *Autonomy outdoors n = 61*, *Social life/Relationships n = 58*, *Work/Education n = 196*

### Predictors of problems experienced at five years after a stroke

The life areas where most problems were experienced due to participation restrictions were mobility, leisure, and helping/supporting other people. We found that older age, severe stroke, and female sex could predict that problems would be experienced in at least one of the affected life areas (Table 5).

**Table 5:** Predictors that problems would be experienced at five years after a stroke.

Predictor models	B (S.E.)	p-value	OR (95% CI)	AUC (95% CI)
**Mobility**				
Stroke severity	1.222 (0.335)	<0.001	3.395 (1.762–6.543)	0.790 (0.733–0.848)
Age at the time of stroke	0.094 (0.015)	<0.001	1.098 (1.066–1.132)	
**Leisure**				
Stroke severity	1.087 (0.309)	<0.001	1.098 (1.066–1.132)	0.733 (0.669–0.797)
Sex	0.897 (0.295)	0.002	2.452 (1.375–4.371)	
Age at the time of stroke	0.042 (0.012)	<0.001	1.043 (1.018–1.067)	
**Helping/supporting others**				
Stroke severity	0.821 (0.308)	0.008	2.272 (1.242–4.156)	0.707 (0.639–0.776)
Age at the time of stroke	0.040 (0.012)	0.001	1.041 (1.017–1.066)	
Sex	0.759 (0.297)	0.011	2.136 (1.193–3.824)	

*Missing data*: *Autonomy indoors n = 62*, *Family role n = 65*, *Autonomy outdoors n = 61*, *Social life/Relationships n = 58*, *Work/Education n = 196*

## Discussion

At five years after a stroke, participation restrictions were most pronounced in the IPA domains of Autonomy Outdoors, Work/Education, and Social Life and Relationships. Conversely, restrictions were less pronounced in the IPA domains of Autonomy Indoors and Family Role. We found that severe stroke, older age, and female sex were predictors of participation restrictions. Feelings of depression at five years after a stroke contributed to the perceived participation restrictions. Problems related to restrictions in participation were most pronounced in the areas of mobility, leisure, and helping/supporting other people; these problems could be predicted by severe stroke, older age, and female sex.

The majority of respondents perceived that their participation and autonomy in life was very good (53%- 72%) at five years after stroke. A minority (5%- 19%) reported poor or very poor participation and autonomy. Accordingly, the standardized mean scores in all of the IPA-domains were low, indicating a very good sense of participation and autonomy. These results differed from other studies that applied the IPA to populations that had experienced stroke [15, 17]; in those studies, the absolute majority of respondents indicated that they perceived good/fair or poor/very poor participation, which reflected higher standardized mean scores for participation restrictions [14, 15, 17]. However, those studies were conducted in different sociocultural environments and healthcare systems, and they collected data at shorter follow-up times after the stroke (3 months to 3 years); thus, the different study conditions might explain the differences in outcomes [15, 17]. Additionally, individuals that have experienced a stroke might acquire a greater acceptance of their situation with time; thus, at five years after the stroke incidence, acceptance could have influenced the perceived participation [27, 28]. It has been suggested that individuals undergo an adjustment process, during the first-year post stroke. In the later stages of this process, the focus is mostly on developing new ways of living with the illness [29, 30]. The majority of persons with stroke in this study had experienced a mild stroke, which also could have influenced their perception of participation restrictions at a later stage [31].

The Autonomy Outdoor domain focused on one’s ability to plan and pursue recreational activities and engage in social events. In previous studies, this domain had been one of the most affected, in terms of restricted participation and autonomy [15, 17, 32], and it had been associated with impaired physical functioning [17]. Physical barriers in the external environment will most likely influence a person’s mobility, and thus, influence the overall sense of participation and autonomy. The domain of Work and Education focused on paid/voluntary work and education; individuals reported on their abilities to engage in work in the way they wanted, take part in a work force, and remain competitive for different job opportunities. The domain of Social Life/Relationships focused on the quality of social life and the individual’s capabilities of establishing and maintaining relationships with friends and acquaintances. Taken together, these three domains comprised aspects of life that required a certain level of physical, social, and cognitive ability. After a stroke, it is common to experience less involvement in physically demanding activities and less satisfaction in recreational, social, and work activities [33]. In turn, these experiences are likely to have consequences on the abilities to engage and participate in activities. For example, restrictions in participation that are commonly experienced by people with stroke in the area of work/occupation [11, 14, 34, 35] could be explained partly by functional disability and partly by emotional status (e.g., depressive symptoms) [11].

In all the IPA domains, except Work and Education, severe stroke and older age at stroke onset were predictors of participation restrictions at five years after the stroke. Previous research has shown conflicting results regarding stroke severity and participation. Stroke severity was found to be negatively associated with participation at one month post stroke [36] and at one year post stroke [37]. However, other studies could not confirm that relationship [38, 39]. Future research is warranted to explore the relationship between stroke severity and participation.

Our finding that older age was associated with more restricted participation was consistent with findings from previous studies [12, 35, 37, 40, 41]. However, restricted participation in daily life and social roles are expected consequences of normal aging [42]; therefore, perhaps part of the perceived participation restrictions could be due to the effects of aging, rather than the effects of stroke, for this population.

In contrast to other studies that assessed participation with the IPA [14, 15, 17], we found that the female sex could predict perceived participation restrictions in the domain of Family Role. Previous studies [43] have shown that traditional gender roles in families are changing slowly. Consequently, women that experience a stroke are less likely to assume that their spouse would provide domestic assistance and act as a potential caregiver; thus, this population was likely to have unmet needs, which might have contributed to the perceived participation restrictions.

At five years after a stroke, in addition to the other predictors, depression contributed to perceived participation restrictions. Post stroke depression is a common condition. It has negative effects on cognition, emotional regulation, social functioning, and it increases the risk of suicide [44–46]. However, the relationship between post stroke depression and functional outcome after stroke is not well understood [45]. Previous studies on self-perceived participation and depression after stroke have shown that depression/depressive moods were associated with restricted participation in various life areas [10, 14, 17, 37, 47]. In terms of the IPA, depressive mood [17] was an explanatory factor associated with restrictions in all the domains. It is possible that the existence of cognitive, functional, and social impairments due to depression could potentially undermine the ability to be active and participate in life situations after a stroke, and thus, impact negatively on the quality of life.

The areas in life that were considered problematic, due to perceived participation restrictions, were Mobility, Leisure, and Helping/supporting other people. The areas of Mobility and Leisure were similar in content, because they involved the perceived ability to move about indoors, visit friends, go on excursions, and maintain leisure activities. We found that perceived problems in these areas could be predicted by a severe stroke, older age at stroke onset, and female sex. This finding was consistent with previous studies that found that restrictions in mobility/physical independence were perceived as problematic, even at several years after a stroke [9, 31]. It was also shown that the perception of a strong ability to perform in the areas of mobility, strength, and self-care was associated with less restrictions in participation at one year post stroke [16].

Social activities and leisure are associated with improved health, satisfaction in life, functional recovery, and survival after stroke [48, 49]. It is believed that leisure encourages people with stroke to feel engaged, with a sense of belonging in a social context [28, 50, 51]. In summary, in the long-term perspective of people with stroke, social activities and leisure are important because they can decrease the risk of social isolation and contribute to a strong sense of engagement and participation in life. Conceivably, mobility and leisure activities could be areas of interest in rehabilitation strategies.

An important goal of rehabilitation treatment is to restore the individual’s participation in society, despite persistent and/or chronic impairments [14, 52]. This study showed that restrictions in participation and autonomy existed among people with stroke at five years after stroke. Domains that required a certain level of physical, social, and cognitive ability were perceived as more restricted after the stroke.

### Limitations

One limitation of this study was that patients were recruited from a stroke unit or a neurosurgical unit at Sahlgrenska University Hospital of Gothenburg, which was the only hospital in Gothenburg that performed neurosurgical treatments, thrombectomies, and thrombolytic procedures. For this reason, younger patients were more often transported to this hospital, which could have resulted in a younger study sample, compared to the general stroke population.

A dropout analysis showed that a significantly larger proportion of women than men did not respond to the questionnaires. In previous studies, female sex was shown to be associated with perceived restrictions in participation [10, 13]. Thus, the large proportion of women dropouts could possibly have influenced the overall results of the IPA-domains.

The respondents in this study, as previously mentioned, had a lower average mean age and a higher percentage of males compared to a general stroke population. Furthermore, the majority of participants had relatively mild strokes. These factors might have reduced the generalizability of our results to a larger stroke population.

### Conclusions

This study showed that, at five years after a stroke, people with stroke experienced restrictions in participation and autonomy. Domains that required certain levels of physical, social, and cognitive abilities were perceived as more restricted. A severe stroke and older age were identified as predictors of perceived participation restrictions in most of the IPA domains. Feelings of depression at five years after stroke also contributed to perceived restrictions. Problems were experienced due to participation restrictions mostly in mobility, leisure, and supporting others. Female sex, severe stroke, and older age increased the risk of experiencing problems in these areas.
